# Factors Affecting Visual Acuity After Anti-Vascular Endothelial Growth Factor Therapy in Neovascular Age-Related Macular Degeneration: A Multicenter Study in Japan

**DOI:** 10.3390/jcm13206244

**Published:** 2024-10-19

**Authors:** Aoi Kominami, Shuhei Tomita, Aki Kato, Koichi Ono, Masaru Takeuchi, Masaya Imazeki, Hiroto Terasaki, Yuki Yamamoto, Tatsuya Jujo, Makiko Wakuta, Hisashi Matsubara, Yoshinori Mitamura, Mineo Kondo, Kazuhiro Kimura, Hitoshi Takagi, Fumi Gomi, Taiji Sakamoto, Tsutomu Yasukawa

**Affiliations:** 1 Department of Ophthalmology and Visual Science, Graduate School of Medical Sciences, Nagoya City University, 1 Kawasumi, Mizuho-cho, Mizuho-ku, Nagoya 467-8601, Japan; pink299pu2pu2@gmail.com (A.K.); yasukawa@med.nagoya-cu.ac.jp (T.Y.); 2 Department of Ophthalmology, Nagoya City University, East Medical Center, 1-2-23 Wakamizu, Chikusa-ku, Nagoya 464-8547, Japan; st_tomita@outlook.com; 3 Department of Ophthalmology, Juntendo Tokyo Koto Geriatric Medical Center, 3-3-20, Shinsuna, Koto-ku, Tokyo 136-0075, Japan; kono@juntendo.ac.jp; 4 Department of Ophthalmology, National Defense Medical College, 3-2 Namiki, Tokorozawa 359-8513, Japan; masatake@ndmc.ac.jp (M.T.); stage271828@gmail.com (M.I.); 5 Department of Ophthalmology, Graduate School of Medical and Dental Sciences, Kagoshima University, Sakuragaoka 8-35-1, Kagoshima 890-8544, Japan; teracchi@m2.kufm.kagoshima-u.ac.jp (H.T.); tsakamot@m3.kufm.kagoshima-u.ac.jp (T.S.); 6 Department of Ophthalmology, Hyogo Medical University, 1-1, Mukogawacho, Nishinomiya 663-8501, Japan; yuki.kom.0923@gmail.com (Y.Y.); fgomi@hyo-med.ac.jp (F.G.); 7 Department of Ophthalmology, School of Medicine, St. Marianna University, 2-16-1 Sugao, Miyamae-ku, Kawasaki 216-8511, Japan; t2jujo@marianna-u.ac.jp; 8 Department of Ophthalmology, Graduate School of Medicine, Yamaguchi University, 1-1-1, Minami-Kogushi, Ube 755-8505, Japan; mwakut@yamaguchi-u.ac.jp (M.W.); k.kimura@yamaguchi-u.ac.jp (K.K.); 9 Department of Ophthalmology, Graduate School of Medicine, Mie University, 2-174 Edobashi, Tsu 514-8507, Japan; hmatsu@med.mie-u.ac.jp (H.M.); mineo@med.mie-u.ac.jp (M.K.); 10 Department of Ophthalmology, Institute of Biomedical Sciences, Graduate School, Tokushima University, 3-18-15, Kuramoto-cho, Tokushima 770-8503, Japan; ymita@tokushima-u.ac.jp; 11 Kawasaki Tama Eye Clinic, 2428, Noborito, Kawasaki 214-0014, Japan; htakagi@kt-eye.com

**Keywords:** anti-vascular endothelial growth factor therapy, multivariate analysis, neovascular age-related macular degeneration, visual prognosis

## Abstract

**Background/Objectives**: Anti-vascular endothelial growth factor (VEGF) therapy is the first-line treatment for neovascular age-related macular degeneration (nvAMD). While proactive and adequate treatment generally leads to better visual outcomes, various factors, including the disease type, ocular findings, lifestyle, and systemic status, affect the visual prognosis in clinical settings. This study aimed to identify the factors that affect the visual prognosis in patients with nvAMD treated with anti-VEGF therapy. **Methods**: We conducted a multicenter retrospective cohort study at eight tertiary referral centers in Japan, where we reviewed the medical records of patients newly diagnosed with nvAMD between January 2014 and December 2019. These patients had started treatment with either ranibizumab (0.5 mg) or aflibercept (2.0 mg) and were followed for at least 1 year. We evaluated the impact of the disease type, systemic factors, and initial fundus findings on the best-corrected visual acuity (BCVA) at 1 year. **Results**: This study included 182 patients (129 men, 53 women), with a mean age of 75.0 ± 8.6 years. The disease types were categorized as typical AMD (53%), polypoidal choroidal vasculopathy (PCV) (43%), and retinal angiomatous proliferation (RAP) (4%). Univariate analysis identified age, the baseline logarithm of the minimum angle of resolution BCVA, intraretinal fluid (IRF), pigment epithelial detachment (PED), and subretinal hyperreflective material (SHRM). Multivariate analysis identified the following significant risk factors associated with vision worsening: age, smoking history, diabetes, and the presence of IRF and PED. **Conclusions**: The presence of IRF, PED, and SHRM at the start of treatment and a history of smoking and diabetes may be associated with a poor visual prognosis in patients with nvAMD.

## 1. Introduction

Neovascular age-related macular degeneration (nvAMD) is a major cause of visual loss in elderly populations in developed countries [1,2,3], and its incidence in Japan has been increasing in recent years [4,5]. Since the efficacy and safety of intravitreal ranibizumab (IVR) (Lucentis, Genentech Inc., South San Francisco, CA, USA) for nvAMD have been demonstrated [6,7], anti-vascular endothelial growth factor (VEGF) therapy is the first-line treatment for nvAMD. Subsequent approvals of aflibercept [8] (Eylea, Regeneron Pharmaceuticals Inc., Tarrytown, NY, USA), brolucizumab [9] (Beovu^®^, Novartis, East Hanover, NJ, USA), faricimab [10] (Vabysmo^®^ Roche, Basel, Switzerland), and aflibercept 8 mg [11] have expanded the anti-VEGF therapeutic options.

Although proactive and sufficient treatments tend to achieve better visual outcomes, many other factors, including the disease type, specific ocular findings, patient lifestyle, or systemic status, affect the visual prognosis in a real-world setting. The development of AMD has long been associated with lifestyle factors such as advanced age [12], supplement intake or dietary balance [13,14,15], smoking [16,17], diabetes, metabolic syndrome or obesity [18,19], hypertension [17], and cardiovascular disorders [20]. Epidemiologic studies in Japan have also reported similar findings [21,22].

Regarding the visual prognosis of nvAMD, follow-up studies from large prospective trials have identified poor prognostic factors. Jaffe et al. [23] evaluated the associations between morphologic features and 5-year visual acuity (VA) in the Comparison of Age-related Macular Degeneration Treatments Trials (CATT) and reported that subretinal hyperreflective material (SHRM), thinner retina, greater choroidal neovascularization lesion area, foveal center pathology, and intraretinal fluid (IRF) were independently associated with worse VA. Sawada et al. [24] reported that the disease type and patient age are also involved in the prognosis. However, few studies have explored the relationship between these risk factors, the initial AMD status at diagnosis, and the visual prognosis in clinical practice [25,26].

The ability to predict the subsequent course of nvAMD at the time of the initial diagnosis in clinical practice could enable more effective guidance on treatment strategies, including the drug selection and treatment regimen. The current study investigated the factors affecting the visual outcomes of nvAMD 1 year after treatment initiation.

## 2. Materials and Methods

### 2.1. Study Design and Ethics

The current study, conducted according to the ethical guidelines of the Declaration of Helsinki, was a multicenter-based case comparison series performed at eight tertiary referral centers in Japan that are members of the Japan Clinical Retina Study Group. The ethics committees of the National Defense Medical College, Nagoya City University, Kagoshima University, Hyogo Medical University, St. Marianna University School of Medicine, Yamaguchi University, Mie University, and Tokushima University approved the study (institutional review board number: 4289). The ethics committees waived the requirement for written informed consent due to the retrospective nature of the study, but all the subjects were informed of the study. An opt-out consent mechanism, as described on the Institutional Review Board-approved website, was available.

### 2.2. Patients

Patients who were newly diagnosed with nvAMD at the eight centers between January 2014 and December 2019 and who had started treatment with ranibizumab 0.5 mg/0.05 mL or aflibercept 2.0 mg/0.05 mL and were followed for at least 1 year were reviewed. Patients without VA data before treatment initiation and at the 1-year follow-up, those with a history of other vitreoretinal diseases such as diabetic retinopathy or branch retinal vein occlusion, patients with uveitis or glaucoma, those with a history of vitrectomy, and those with a history of anti-VEGF therapy were excluded.

### 2.3. Research and Analysis

This study investigated the factors affecting the best-corrected VA (BCVA) 1 year after the initiation of treatment for nvAMD. The factors related to the BCVA in the affected eye after 1 year were considered as follows: the variables were selected based on systemic factors identified in previous studies, such as the Age-Related Eye Disease Study [17] and Funagata Study [22], with anatomic findings explored in reference to the CATT Study results [23]. The treatment protocols followed the guidelines of the Japanese Ophthalmological Society [27].

**Figure 1:**
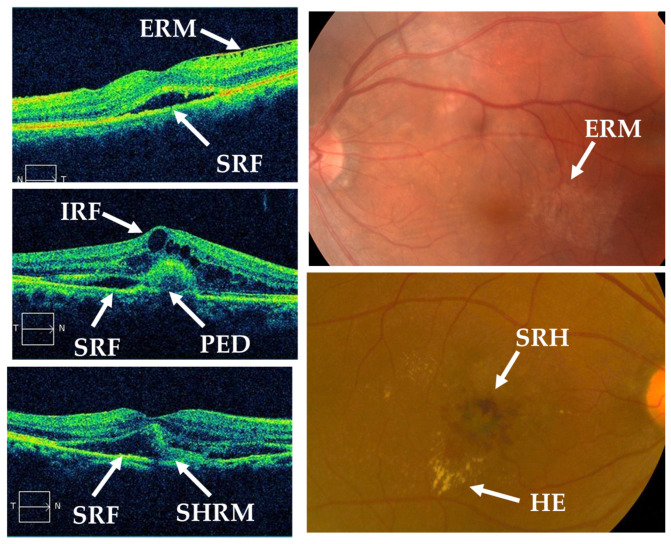
Optical coherence tomography images and color fundus photographs. The arrows indicate IRF, SRF, PED, SHRM, SRH, HE, and ERM.

**Figure 2:**
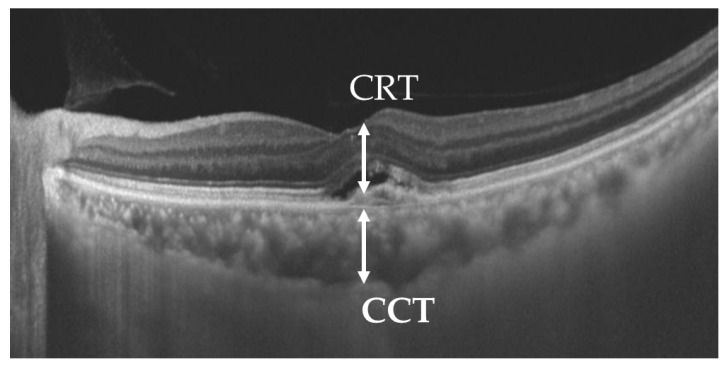
Measurement of the CRT and CCT. CRT: from the internal limiting membrane to the retinal pigment epithelium. CCT: from the outer surface of the retinal pigment epithelium to the inner sclera.

1. Patient background factors: age, sex, AMD subtype (polypoidal choroidal vasculopathy [PCV], typical AMD [tAMD], or retinal angiomatous proliferation [RAP]), smoking history, diabetes, hypertension (HT), cardiovascular disease (CAVD), cerebrovascular disease (CEVD), and use of anticoagulants.
2. Optical coherence tomography (OCT) and fundus findings: the presence or absence of subretinal fluid (SRF), intraretinal fluid (IRF), pigment epithelial detachment (PED), subretinal hyperreflective material (SHRM), epiretinal membrane (ERM), hard exudates (HE), and subretinal hemorrhage (SRH) was evaluated based on OCT images and fundus examination (Figure 1).

3. Thickness measurements: the central retinal thickness (CRT) and central choroidal thickness (CCT) were measured either automatically or manually using OCT depending on the imaging system (Figure 2). The CRT was measured from the internal limiting membrane to the retinal pigment epithelium, and the CCT was measured from the outer surface of the retinal pigment epithelium to the inner sclera.

4. Treatment-related factors: the type of drug (aflibercept or ranibizumab), the treatment regimen (pro re nata, modified treat and extend [modified TAE], TAE, or bimonthly), any changes in treatment (no change, drug switch, or combined with photodynamic therapy [PDT]), the number of intravitreal injections, and whether the treatment was maintained over the course of 1 year.

### 2.4. Statistical Analysis

The BCVA was analyzed by converting the values from decimal units to the logarithm of the minimum angle of resolution (logMAR) units. A history of CAVD and CEVD was treated as part of the composite outcome of cardiovascular disease (CVD). The age, logMAR BCVA, CRT, and CCT were treated as continuous variables, while the other factors were treated as categorical variables. Only patient information without missing values obtained from each facility was analyzed. Statistical analysis was performed using univariate regression analysis to investigate the relationship between the logMAR BCVA at 1 year and each factor. Statistical comparisons among the three groups (PCV, tAMD, and RAP) were performed. Continuous variables were analyzed using analysis of variance, and categorical variables were analyzed using the chi-square test. For variables exhibiting significant differences, post hoc tests were conducted to identify the specific groups that differed from one another. Paired *t*-tests also were used to assess the changes in the logMAR BCVA from baseline to the 1-year follow-up, comparing the overall AMD group and each subtype. Given that this study was exploratory, multivariate regression analysis was conducted using factors with a *p* value of less than 0.25. A *p* value less than 0.05 was considered statistically significant. All the statistical analyses were conducted using Stata version 15.1 (StataCorp, College Station, TX, USA).

## 3. Results

### 3.1. Patient Characteristics

Data were obtained from 630 participants across eight Japanese facilities. Among these, 182 participants (129 men, 53 women) with complete data were included in the analysis, representing 182 eyes (78 with PCV, 97 with tAMD, and 7 with RAP). The mean age ± standard deviation (SD) of the subjects was 75.0 ± 8.6 years (range, 50–94 years). Table 1 shows the background and treatment details of all the subjects and those categorized by AMD type. No significant differences were observed among the AMD types for all the variables except the treatment regimen.

### 3.2. Baseline CRT and CCT

**Figure 3:**
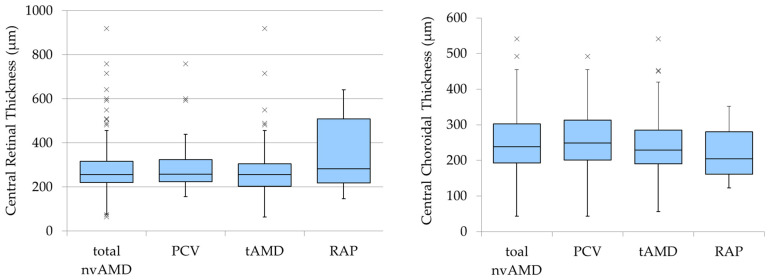
The box plots compare the CRT and CCT in all the patients and by AMD subtype before treatment. No significant differences were observed among the groups for CRT or CCT.

The mean CRT ± SD before treatment was 283.2 ± 103.0 μm for PCV, 273.1 ± 119.2 μm for tAMD, and 360.6 ± 189.7 μm for RAP, with no significant differences among the three groups (F(2,179) = 1.89, *p* = 0.154). Similarly, the mean CCT ± SD was 260.3 ± 85.0 μm for PCV, 244.5 ± 83.2 μm for tAMD, and 223.1 ± 85.3 μm for RAP, with no significant differences among the three groups (F(2,179) = 1.15, *p* = 0.319) (Figure 3).

### 3.3. Changes in BCVA

**Figure 4:**
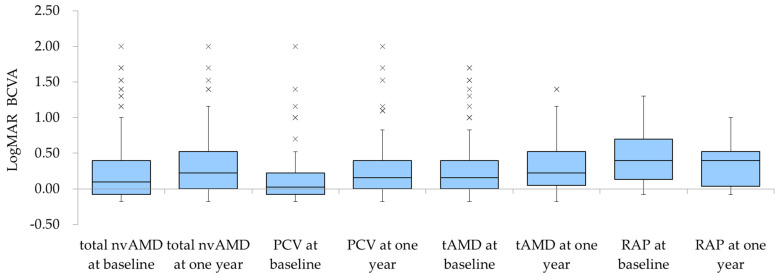
The changes in the logMAR BCVA before and 1 year after treatment for PCV, tAMD, or RAP. Significant changes were seen in the overall logMAR BCVA (*p* = 0.0176). Stratified analysis indicated no significant changes in the logMAR BCVA in each group (PCV, tAMD, RAP) and no significant differences in the logMAR BCVA among the disease types before treatment and at 1 year.

Figure 4 shows the changes in the logMAR BCVA before and 1 year after treatment. A significant difference was seen in the logMAR BCVA, which increased from 0.242 ± 0.418 to 0.331 ± 0.423 (*p* value: 0.0176) Although statistically significant, this change was not clinically significant. The stratified analysis showed that the logMAR BCVA changed from 0.171 ± 0.378 to 0.281 ± 0.437 for PCV, from 0.283 ± 0.437 to 0.371 ±0.414 for tAMD, and from 0.469 ± 0.473 to 0.349 ± 0.385 for RAP, with no significant differences in any of these groups (*p* = 0.0601, 0.0944, and 0.217, respectively). No significant differences in the logMAR BCVA were seen between the disease types before the start of treatment and at 1 year (*p* = 0.07 and 0.376, respectively).

### 3.4. Association of Each Parameter with BCVA at 1 Year

Univariate regression analysis showed that age (0.012; 95% confidence interval [CI]: 0.005–0.019), baseline logMAR BCVA (0.288; 95% CI: 0.145–0.431), and OCT findings of IRF (0.227; 95% CI: 0.088–0.366), PED (0.136; 95% CI: 0.012–0.259), and SHRM (0.131; 95% CI: 0.006–0.257) resulted in worse vision. In an exploratory multivariate analysis model that included factors with *p* < 0.25, age (0.010; 95% CI: 0.002–0.017), smoking history (0.164; 95% CI: 0.042–0.286), diabetes (0.214; 95% CI: 0.060–0.369), IRF (0.187; 95% CI: 0.044–0.330), and PED (0.145; 95% CI: 0.022–0.268) on OCT were significant risk factors (Table 2).

### 3.5. Association Between CRT and OCT/Fundus Findings

Table 3 shows the relationship between the CRT and OCT findings. Univariate and multivariate analyses showed no association between the presence of IRF (0.321; 95% CI: −19.4–58.7) or PED (0.461; 95% CI: −47.5–21.6) and CRT. ERM (0.002; 95% CI: 65.9–292.3) was the only factor significantly associated with CRT.

## 4. Discussion

This study investigated the factors affecting VA in patients with nvAMD 1 year after the start of treatment. The distribution of AMD subtypes in our cohort was consistent with previous reports [28,29], i.e., 43% PCV, 53% tAMD, and 4% RAP. While there were no significant differences in the patient histories among the different AMD subtypes, bimonthly dosing was prescribed more frequently for patients with RAP compared to the other subtypes. Notably, no significant differences in VA were observed among the subtypes before or after treatment, and the VA was maintained across all the subtypes at the 1-year follow-up. Regarding the systemic factors, univariate analysis showed a correlation between visual prognosis and age. Multivariate analysis further identified age, smoking, and a history of diabetes as poor prognostic factors for VA. The ophthalmic findings at the start of treatment also played a critical role in the visual outcomes. Univariate analysis showed that the presence of IRF, PED, and SHRM was correlated with VA at 1 year. Multivariate analysis found that the presences of IRF and PED were poor prognostic factors for VA. In addition, the pretreatment CRT was associated with the presence of ERM in the univariate analysis, with ERM was identified as a factor that worsened the CRT in the multivariate analysis.

We observed a trend in which the CCT was thickest in PCV, followed by tAMD, and thinnest in RAP; however, these differences did not reach significance. It is well established that PCV, as part of the pachychoroid spectrum [30], is associated with thicker CCT [31,32], whereas RAP generally is associated with thinner CCT [33,34]. The lack of significant differences in our study may be attributed to several factors. First, we only included cases in which anti-VEGF therapy was the initial treatment. Consequently, PCV cases with prominent pachychoroid features might have been treated with PDT monotherapy, potentially skewing the results. Second, the small number of RAP cases may have reduced the statistical power, making it difficult to detect significant differences. Considering these factors, further research may be necessary to draw definitive conclusions regarding the CCT thickness.

Regard VA, the current cases were newly diagnosed with AMD during the study period. The mean logMAR BCVA before treatment was 0.242, which corresponds to about 75 letters on the Early Treatment Diabetic Retinopathy Study chart. This indicates that many of the current patients had better baseline VA compared to those reported in larger prospective studies. While there was a significant difference between the pre- and post-treatment BCVAs in the overall cohort, it is important to note that clinical significance was typically defined by a change of 0.3 logMAR or more. Therefore, despite the significance, the VA changes in this study were not clinically relevant [35], leading us to conclude that the VA was effectively maintained over the course of 1 year. Consistent with previous studies [12], our findings confirmed that older age was associated with poorer visual prognoses in patients with nvAMD.

A key focus of our investigation was the relationship between the patient history and the 1-year visual outcomes. While smoking is a well-documented risk factor for AMD development [16,17], Lee et al. reported [25] that smoking is an independent risk factor for lower VA gains with IVR treatment for exudative AMD, and our results suggested that it may also adversely affect the prognosis after the start of treatment. The link between smoking and visual prognosis in AMD has not been fully elucidated, but several potential mechanisms have been proposed. Smoking is associated with increased oxidative stress, lipid peroxidation, elevated fibrinogen levels, and enhanced platelet aggregation, and it is correlated with decreased levels of high-density lipoprotein and antioxidants in plasma [36]. Further, nicotine-induced up-regulation of VEGF may promote neovascularization [37,38]. Smoking also activates complement component C3 and other inflammatory mediators while reducing the serum levels of complement factor H, thereby exacerbating inflammation [36]. These factors suggest that smoking may directly affect the choroidal circulation, leading to reduced choroidal blood flow [39], and may decrease the lutein and zeaxanthin levels in the macular retina, exposing this tissue to more severe oxidative damage. These mechanisms not only contribute to the onset of AMD but may also affect its progression and potentially diminish the effectiveness of anti-VEGF therapy.

Diabetes was another factor impacting the visual prognosis in our study. Recent studies have highlighted the significance of diabetes and diabetic retinopathy in the context of AMD and the efficacy of anti-VEGF therapy. Topouzis et al. [40] reported a positive association between diabetes and the development of nvAMD, particularly in patients with early or dry AMD. Further, Boscia et al. [41] found that in patients with nvAMD, those with diabetes had a significantly lower reduction in the macular neovascularization (MNV) area during anti-VEGF treatment, as assessed by OCT angiography (OCTA). Viggiano et al. [42], who investigated the morphologic modifications of the choriocapillaris using OCTA in patients with type 1 MNV before and after the introduction of intravitreal anti-VEGF agents, observed that patients with diabetes had significantly lower choriocapillaris perfusion even before treatment compared to patients without diabetes, and this did not significantly change after treatment. This finding suggests that diabetes should be considered a risk factor in patients undergoing anti-VEGF therapy for nvAMD. As the number of patients with concurrent diabetes and nvAMD is expected to rise, further investigation into this association is warranted to optimize the treatment outcomes in this growing patient population.

Another key focus of this study was the relationship between anatomic findings and visual prognosis. In the univariate analysis, the presence of SHRM was correlated with visual prognosis, while both PED and IRF were not only correlated with visual outcomes but were also significant factors in the multivariate analysis. PED is often refractory to anti-VEGF therapy [43,44], and when complicated by a retinal pigment epithelial (RPE) tear, can lead to a significant visual decline due to subsequent retinal atrophy [45]. Recently, Sarraf et al. [46] reported that greater PED thickness and PED thickness variability are associated with poorer visual outcomes in patients with nvAMD. Our study was conducted between 2014 and 2019, when only ranibizumab (0.5 mg) and aflibercept (2 mg) were used. However, newer agents, such as the recently approved brolucizumab and faricimab, are more effective against PEDs [47,48,49]. Future research is needed to evaluate these newer treatments.

The presence of IRF and SHRM predicts poor VA [23,43,50,51]. IRF associated with type 2 MNV, in which the neovascularization is above the RPE, and type 3 MNV, in which the neovascularization is within the retina, can directly damage the retina, leading to rapid visual decline. Regarding SHRM, Kumar et al. [52] reported that “increased width and area of SHRM at the foveal center at baseline was correlated with visual deterioration at week 24”. We previously reported that early resolution of SHRM is associated with maintained VA [53]. Other studies found that brolucizumab is effective against SHRM [54], similar to its effects on PED, suggesting the need for further investigation. Other factors, such as HE and SRH, are considered markers of activity in nvAMD, particularly in PCV, but in our study, they were not directly related to the visual prognosis. Our study also found a strong correlation between the ERM and CRT. In large-scale prospective studies, cases with ERM, which can affect VA, are often excluded from the inclusion criteria. However, in real-world settings, ERM frequently coexists with AMD. While ERM affect the CRT, the visual prognosis does not seem to be affected significantly, as indicated by our findings. This insight could be valuable for guiding future treatment strategies.

This study has several limitations. First, as a retrospective observational study, there was no standardized protocol for the selection of treatment drugs, the use of PDT, or the treatment regimens. This study was conducted across multiple centers, which may have led to variability in the regimen selection and retreatment criteria. For instance, in our analysis, bimonthly dosing was more frequently chosen in RAP cases compared to the other AMD subtypes. This may be due to the high recurrence rate of RAP, leading clinicians to prefer a fixed-dosing regimen during the first year of treatment. Second, regarding the evaluation of the patient history, this study only considered the presence or absence of certain conditions and did not assess the current status of these factors. Specifically, we did not evaluate the smoking status. Since there is a dose–response relationship for smoking, and evidence suggests that the risks may be reversible with cessation [55], a more detailed investigation is warranted. This study also did not examine the impact of supplement intake or alcohol consumption. Supplements reduce the onset of disease in the fellow eye [13,14]; additionally, a follow-up study by the ARED reported that supplements containing lutein and omega-3 suppressed the development of nvAMD [56]. Furthermore, attention has recently also been paid to the effects of carotenoids such as astaxanthin and fucoxanthin, the trace element selenium, the polyphenol curcumin, and the neurohormone melatonin in the treatment of AMD [57,58]. Alcohol consumption has also been implicated as a potential risk factor [59,60]. However, the impact of these lifestyle factors on the treatment outcomes may not be fully apparent within the 1-year follow-up period, and longer studies are needed to better understand their effects. The third limitation of this study is that the post-treatment parameters were limited to VA. Other important anatomic outcomes, such as CRT and the presence or absence of SRF, IRF, PED, and SHRM, were not assessed after treatment. Future studies should include these parameters to provide a more comprehensive evaluation of the treatment outcomes.

Despite these limitations, our study provides valuable insights. We confirmed that systemic factors such as smoking and diabetes not only contribute to the risk of developing nvAMD but also adversely affect the visual prognosis. Moreover, anatomic features such as IRF, PED, and SHRM were identified as poor prognostic factors. The presence of these features before treatment initiation was associated with worse visual outcomes, which is a significant finding.

## 5. Conclusions

In this retrospective study of patients with nvAMD, we identified several factors that affect the visual prognosis after 1 year of treatment. Older age, smoking, and diabetes were significant systemic predictors of poor visual outcomes. Anatomic features such as IRF, PED, and SHRM were also associated with a worse visual prognosis, particularly when present before treatment initiation. Despite the study’s limitations, including the variability in treatment regimens and the lack of detailed assessments of lifestyle factors, these findings underscore the importance of considering both systemic and anatomic factors in the management of nvAMD.

## Figures and Tables

**Table 1:** Patient characteristics and treatment.

		Total (*n* = 182)		PCV (*n* = 78)		tAMD (*n* = 97)		RAP (*n* = 7)		*p* Value
Age (years)	Mean ± SD	75.0 ± 8.6		74.0 ± 8.7		75.6 ± 8.6		78.3 ± 8.0		0.277
	Range	50–94		54–94		50–91		7–87		
Sex	Men	129	70.9%	58	74.4%	66	68.0%	5	71.4%	0.658
	Women	53	29.1%	20	25.6%	31	32.0%	2	28.6%	
Smoking	Yes	107	58.8%	45	57.7%	58	59.8%	4	57.1%	0.957
DM	Yes	34	18.7%	15	19.2%	18	18.6%	1	14.3%	0.949
HT	Yes	103	56.6%	44	56.4%	55	56.7%	4	57.1%	0.999
CVD (CAVD and/or CEVD)	Yes	28	15.4%	15	19.2%	12	12.4%	1	14.3%	0.456
Anticoagulant	Yes	29	15.9%	12	15.4%	16	16.5%	1	14.3%	0.973
Treatment										
Type of anti-VEGF	Aflibercept	123	67.6%	50	64.1%	70	72.2%	3	42.9%	0.191
	Ranibizumab	59	32.4%	28	35.9%	27	27.8%	4	57.1%	
Regimen	PRN	84	46.2%	35	44.9%	45	46.4%	4	57.1%	<0.001 ^†^
	Modified TAE	6	3.3%	0	0.0%	6	6.2%	0	0.0%	
	TAE	88	48.4%	42	53.8%	45	46.4%	1	14.3%	
	Bimonthly	4	2.2%	1	1.3%	1	1.0%	2	28.6%	
Switch	No	147	80.8%	59	75.6%	82	84.5%	6	85.7%	0.353
	Anti-VEGF	21	11.5%	13	16.7%	7	7.2%	1	14.3%	
	PDT	14	7.7%	6	7.7%	8	8.2%	0	0.0%	
PDT combined with initial treatment	Yes	7	3.8%	4	5.1%	2	2.1%	1	14.3%	0.197
Continuity *	Yes	169	92.9%	71	91.0%	91	93.8%	7	100.0%	0.587
No. injections	Mean ± SD	5.4 ± 2.4		5.6 ± 2.38		5.2 ± 2.4		5.0 ± 2.1		0.887
	Range	1–3		1–11		1–13		2–8		

The % agreement between CVD and anticoagulant was 92.86% (kappa: 0.7296). PRN = pro re nata; DM = diabetes mellitus. ^†^ Post hoc test results: PCV vs. RAP; *p* < 0.001, tAMD vs. RAP; *p* < 0.001, PCV vs. tAMD; no significant difference. * Continuity: refers to the proportion of patients who continued treatment throughout the year.

**Table 2:** Univariate regression analysis and multivariate analysis.

			Simple Regression Model				Multivariate Regression Model			
			Coef.	(95% Interval)		*p* > t	Coef.	(95% Interval)		*p* > t
Background	Age (years)		0.012	0.005	0.019	0.001	0.010	0.002	0.017	0.011
	Sex	Men vs. women	0.023	−0.114	0.159	0.742				
	AMD subtype	PCV	0	-	-	-	0	-	-	-
		tAMD	0.090	−0.037	0.217	0.164	0.093	−0.028	0.213	0.130
		RAP	0.068	−0.262	0.397	0.685	−0.128	−0.453	0.196	0.436
	Smoking	Yes vs. no	0.111	−0.014	0.236	0.081	0.164	0.042	0.286	0.009
	DM	Yes vs. no	0.155	−0.003	0.312	0.054	0.214	0.060	0.369	0.007
	HT	Yes vs. no	0.074	−0.051	0.199	0.243	−0.074	−0.200	0.052	0.249
	CVD (CAVD + CEVD)	Yes vs. no	0.128	−0.043	0.299	0.140	0.047	−0.117	0.211	0.573
	Anticoagulant	Yes vs. no	0.060	−0.109	0.230	0.484				
OCT findings	SRF	Yes vs. no	0.016	−0.178	0.210	0.869				
	IRF	Yes vs. no	0.227	0.088	0.366	0.001	0.187	0.044	0.330	0.011
	PED	Yes vs. no	0.136	0.012	0.259	0.032	0.145	0.022	0.268	0.021
	SHRM	Yes vs. no	0.131	0.006	0.257	0.041	0.021	−0.103	0.146	0.735
	ERM	Yes vs. no	0.192	−0.231	0.614	0.371				
	HE	Yes vs. no	0.116	−0.013	0.246	0.078	0.034	−0.092	0.159	0.597
	SRH	Yes vs. no	0.101	−0.027	0.229	0.121	0.078	−0.043	0.199	0.203
	CRT		0.000	0.000	0.001	0.195	0.0004	−0.0002	0.001	0.190
	CCT		−0.001	−0.001	0.000	0.106	−0.0002	−0.001	0.001	0.654
Intervention	Anti-VEGF	A vs. R	0.086	−0.045	0.218	0.198	0.106	−0.022	0.234	0.105
	regimen	PRN	0							
		modified TAE	−0.158	−0.511	0.196	0.380				
		TAE	0.067	−0.060	0.195	0.298				
		bimonthly	0.091	−0.337	0.519	0.676				
	Switch	No	0							
		Anti-VEGF	−0.016	−0.212	0.179	0.869				
		PDT	0.061	−0.173	0.296	0.607				
	Frequency	Yes vs. no	−0.017	−0.043	0.009	0.205	−0.012	−0.037	0.012	0.323
	PDT	Yes vs. no	0.039659	−0.28302	0.362337	0.809				
	continuity	Yes vs. no	−0.076	−0.316	0.165	0.536				
LogMAR BCVA at baseline			0.288	0.145	0.431	0.000	0.153	−0.006	0.311	0.059

DM = diabetes mellitus; HT = hypertension; A = aflibercept; R = ranibizumab.

**Table 3:** Measure of association between CRT and OCT/fundus findings.

	Simple Regression Model				Multivariate Regression Model			
	Coef.	(95% Conf.	Interval)	*p* > t	Coef.	(95% Conf.	Interval)	*p* > t
SRF	46.4	−6.6	99.3	0.086	38.4	−13.7	90.5	0.148
IRF	30.6	−8.4	69.6	0.123	19.7	−19.4	58.7	0.321
PED	−8.6	−42.9	25.8	0.623	−12.9	−47.5	21.6	0.461
SHRM	33.3	−1.4	67.9	0.060	32.7	−3.0	68.4	0.073
ERM	192.2	79.3	305.0	0.001	179.1	65.9	292.3	0.002
HE	2.7	−33.2	38.5	0.884	−2.3	−39.3	34.6	0.902
SRH	2.0	−33.4	37.4	0.911	−8.2	−43.5	27.0	0.645

## Data Availability

Researchers can contact Aki Kato, MD, PhD, (akikato@med.nagoya-cu.ac.jp) for details of the protocol and results.
